# Transesterification in Microreactors—Overstepping Obstacles and Shifting Towards Biodiesel Production on a Microscale

**DOI:** 10.3390/mi11050457

**Published:** 2020-04-28

**Authors:** Martin Gojun, Matea Bačić, Anabela Ljubić, Anita Šalić, Bruno Zelić

**Affiliations:** Faculty of Chemical Engineering and Technology, University of Zagreb, Marulićev trg 19, HR-10000 Zagreb, Croatia

**Keywords:** lipase catalyzed transesterification, biodiesel, microreactors, deep eutectic solvents

## Abstract

Biodiesel, which was earlier used only as an alternative fuel, is now an indispensable component of commercial diesel. Conventional production processes are unable to cope with the increasing demand for biodiesel, and therefore more and more work is being done to intensify the existing processes. The intensification of the biodiesel production process, taking into account the environmental and economic factors, is based on increasing productivity. One way to achieve that is by reducing the volume of production units. The application of the enzymatic reaction path, while reducing the volume of process equipment to the micro-level, has significantly magnified the productivity of the biodiesel production process, which is primarily due to better mass transfer in microsystems. Additional breakthrough is the use of deep eutectic solvents (DES) instead of buffers for enzyme stabilization. In this study, a lipase from *Thermomyces lanuginosus* (TlL) (both commercial and produced by solid-state fermentation) was used as a catalyst for biodiesel production. Edible and waste sunflower oil, as well as methanol, were used as substrates. The reaction mediums were buffer and DES. The transesterification reaction was carried out in a batch reactor and the emphasis was made on different microreactor configurations. The highest yield of 32% for residence time of only *τ* = 30 min was obtained in the microreactor system with an emulsion of waste oil and a commercial enzyme suspended in a buffer. This indicates that enzymatic transesterification could be a valuable reaction path for dealing with waste oils. Furthermore, biodiesel synthesis in DES showed somewhat lower yields, but by increasing the water content in the system, the reaction could prove much better results. In the end, the effects of reaction conditions on the volumetric productivity of the process were analyzed.

## 1. Introduction

Biodiesel, a mixture of fatty acid methyl esters (FAME), is a biodegradable and non-toxic alternative fuel to petrol diesel. When compared to fossil biodiesel, it excels in its bio-degradability, minimal toxicity and a near zero-emission of aromatic compounds, sulfates and other chemical components which have a negative effect on the environment [1,2,3].

The usage of alternative fuels emerged more than a hundred years ago, when Rudolf Diesel used vegetable oil as fuel in his engine [4]. During the first part of the 20th century, vegetable oils were used instead of diesel fuels only occasionally. Until the mid-1970 s, alternative and renewable fuel sources had hardly any economic and ecological impact. Due to climate change, mostly comprising of air pollution and global warming caused by CO_2_ and declining petroleum reserves (which imminently leads to an increase in crude oil prices), the development of alternative fuel sources got full attention, was backed up by the government, the general public, researchers and industries [5]. Biodiesel has been identified as one of the most prominent options for partially reducing the use of conventional fossil fuels, which resulted in the fact that 60% of new cars and trucks operating in Europe run on biodiesel [6]. State of the art internal combustion engines can use pure B100 biodiesel (100% biodiesel), but the more common use in Europe is a mixture of fossil diesel and biodiesel with the most common being B7 (7% biodiesel) [7].

Industrial production of biodiesel can be carried out by four methods: blending, micro-emulsification, pyrolysis and transesterification [8]. Biodiesel, regardless of the production method, must meet the quality standards according to EN 14,214 [9]. Each of the biodiesel production methods has its advantages and disadvantages and transesterification has proven to be the most efficient and the most economical method. The transesterification process path can be carried out as homogeneous or heterogeneous, according to the nature of the catalyst. Furthermore, based on the nature of the catalyst, transesterification can be chemical or enzymatic [10]. Different oils and fats of vegetable and animal origin can be used in the transesterification process and the thing that makes this process particularly acceptable in terms of environmental impact is the possibility of using waste oils and fats [11].

The use of enzymes in industrial processes is increasingly prevailing. One of the most represented enzymes in the industry is lipase (EC 13.1.1.3.). Lipase belongs to the group of hydrolases and can simultaneously catalyze a number of reactions such as transesterification, hydrolysis and esterification [6,12]. Although lipase enzymes from yeast of the genus Candida are the most represented in research and are used in various fields, ranging from the production of low-energy chemicals to biodiesel [11], some authors cite lipases from *Thermomyces lanuginosus* (TlL) as very effective catalysts [10]. TlL lipases are basophilic and thermostable enzymes and are commercially available as suspensions or in an immobilized form. One of the processes in which TlL lipases have found application is the production of biodiesel, where the high stability of TlL has a significant impact on process efficiency [9]. Since it catalyzes the transesterification reaction, the use of lipase has recently been increasing for biodiesel production. Several reasons can be pointed out: the use of edible and waste oils as raw materials, whereby waste oils can be used without pre-treatment; the transesterification reaction is carried out under mild process conditions. Furthermore, the lipase can be reused in the process because it is easy to separate other components of the reaction mixture, which makes the whole biodiesel production process more efficient, both economically and environmentally. Glycerol produced as a by-product is of high purity and can be used as a feedstock in other processes without further processing [6,13].

Given the increasing demand for biodiesel, there is a need to improve existing conventional production processes in order to catch up with the needs of the market. Therefore, biodiesel batch production processes are recently being replaced by continuous processes using heterogeneous catalysts to intensify the process and carry it out with a small number of process steps, which leads to a small amount of waste process streams. Microreactors have been recognized as one of the most significant technologies that usually result in process intensification and among other processes in which they are used, they have also shown their applicability in biodiesel production [14].

Microreactors are reactor systems on a microscopic scale that have been produced, partially or completely, using microtechnology and microengineering [15]. A microreactor consists of a microchannel network of the usual diameter of 10–500 µm. The small dimensions of the microchannels enable efficient mass and energy transfer, which, with a short retention time, contributes to the intensification of the conducted processes. The small size of a microreactor leads to a small amount of waste process streams and lower energy consumption, since it involves the use of small quantities of reactants and catalysts. In addition, microreactors have some other advantages, of which the most important ones are compactness and ease of implementation, the laminar flow of process streams, efficient mixing and the short diffusion path of the molecules [16]. The aforementioned characteristics of microreactors also give actual reasons for their use, which include the high utilization and productivity achieved during the implementation of the process, including safe working conditions [11,17]. Additional distinction, as well as an advantage, of microreactors is the scale-up of the conducted processes. In comparison to the classical scale-up approach, the process steps in microreactors are magnified by connecting the process units in parallel or in series [18]. Thus, the long and costly approach to scale-up process characteristic for meso- and macro-reactors can simply be overcome by connecting individual microreactors. In that way, the overall capacity of the process is increased and the characteristics of each microreactor that make up such a system remain the same. Despite all of the aforementioned advantages of microreactor systems, their use for carrying out enzymatically catalyzed reactions is not as widely represented in the literature [19].

Despite all of the aforementioned advantages, the commercialization of the enzymatic process for biodiesel production faced a major obstacle—the high cost of the enzyme and methanol inhibition [20]. One possible way to tackle this obstacle is the usage of deep eutectic solvents (DES) as reaction mediums. They have been suggested because of their enhancement of mass transfer, as well as the reduction of glycerol accumulation and methanol inhibition. Furthermore, they are prepared in mild reaction conditions and from cheap substrates [21].

In this study, a TlL (both commercial and produced by solid-state fermentation) was used as a catalyst for biodiesel production. Edible sunflower oil and waste oil were raw materials used for biodiesel production coupled with methanol, while reaction mediums were buffer and DES. Different experiments were set up, including both batch processes and microreactor experiments, respectively. In all the experiments, FAME yield was monitored as the most important indication for experimental set up, which could be further explored. In the end, the comparison of biodiesel synthesis within different types of microreactors and in a batch reactor was given. Based on yield and volumetric productivity, the best system for biodiesel production was proposed.

## 2. Materials and Methods

### 2.1. Materials

#### Chemicals

Edible sunflower oil (Zvijezda, Zagreb, Croatia) was bought in a supermarket. Waste cooking oil (WCO) used in this research was collected after deep frying of potatoes. F.A.M.E. mix GLC-10, the commercial lipase from *Thermomyces lanuginosus* (Lipolase 100L), isoamyl alcohol, iso-octane and sodium dodecyl sulfate (SDS) were purchased from Sigma-Aldrich Handels GmbH (Vienna, Austria). Chloroform and acetonitrile were purchased from Fisher Chemicals (Loughborough, United Kingdom). Tris (hydroxymethyl) aminomethane (TRIS), methanol, HCl and *n*-heptane were purchased from BDH Prolabo (VWR, London, United Kingdom). Dipotassium hydrogen phosphate (K_2_HPO_4_) and ammonium sulfate were purchased from Merck (Darmstadt, Germany). KOH and potassium dihydrogen phosphate (KH_2_PO_4_) were purchased from Lach:ner (Prague, Czech Republic). Sodium hydrogen carbonate was purchased from Kemika (Zagreb, Croatia). 4-nitrophenyil-acetate was purchased from Acros Organics (Fischer Scientific, Geel, Belgium).

### 2.2. Methods

#### 2.2.1. Lipase Production and Purification

Solid-state fermentation of by-products from cold-pressing oil production was used for the production of lipase from fungi *Thermomyces lanuginosus.* Lipase production and purification is described elsewhere [22].

#### 2.2.2. Lipase Assay

Enzyme activity was determined by a test described elsewhere [23]. Only difference was use of 0.0375 mol/L 4-nitrophenyl acetate in assay. Briefly, 100 μL of the sample was added to 3900 μL of 50 mmol/L TRIS-HCl buffer pH 8 and homogenized. 1 mL is of sample is needed to start the test; 950 μL of previously made mixture was added to UV-cuvette and reaction was started with the addition of 50 μL of 0.0375 mol/L 4-nitrophenyl acetate (dissolved in acetonitrile). For determination of the enzyme activity, spectrophotometer (Shimadzu UV–1601, Kyoto, Japan) was used. The change of absorbance was measured at 400 nm, with the total determination time of 20 s. To confirm repeatability, all measurements were performed in triplicate. The results showed no significant difference on 95% confidence interval.

#### 2.2.3. Emulsion Preparation

The preparation of water-in-oil emulsion started with addition of buffer (containing the enzyme) to oil in 8:1 ratio, followed by the addition of SDS as the selected emulsifier (*γ* = 0.1 mg/mL). The mixture was then mixed on the laboratory shaker (Tehtnica, Vibromix 313EVT, Prague, Czech Republic) at 600 rpm for 25 min.

#### 2.2.4. Deep Eutectic Solvent (DES) Preparation

Anhydrous DES used in this work was the combination of choline chloride (20.16 g) and glycerol (38.88 g) in a molar ratio of 1:3.0 [21]. After weighing, the above-mentioned masses were placed in a beaker and stirred on a magnetic stirrer (200 rpm) at 50 °C. The process was carried out for approximately 60 min until a homogeneous, colorless and transparent liquid (*T* = 25 °C, *ρ* = 1.2250 g/mL, *η* = 0.078 Pa s) is obtained. DES was then cooled to room temperature and stored.

#### 2.2.5. Determination of Free Fatty Acids (FFA) in Oil (Chemical Synthesis)

Concentration of FFA in sunflower oil, was determined by dissolving edible sunflower oil (60 mg) in 4 mL of iso-octane, after which 200 µL of 2 mol/L KOH methanol solution was added. Mixture was rapidly shaken for 30 s and then left on room temperature. When the mixture became transparent and with visible layer of glycerol decanted at the bottom of the flask, 1 g of sodium hydrogen carbonate was added in order to neutralize the mixture. In order to determine FFA, upper layer of the mixture was separated and analyzed by gas chromatography, applying the same method which is briefly described in Section 2.2.6.

#### 2.2.6. Measurement of Fatty Acid Methyl Esters (FAME) and Glycerol Concentrations

FAME and glycerol concentration were determined according to the method described by Budžaki et al. [24] on a gas chromatograph (Shimadzu GC-2014, Tokyo, Japan) equipped with FID and Zebron ZB-wax GC capillary column (length 30 m, I.D. 0.53 mm and film thickness 1.00 μm, Phenomenex, Torrance, CA, USA). Carrier gas in this method was nitrogen, at rate of 1.97 mL/min. With the total determination time of 15 min, measurement starts at the temperature of 180 °C for 1 min, with column heating up to 230 °C, at rate of 5 °C/min. F.A.M.E. mix GLC-10 was used as a standard for identifying peaks for corresponding esters of fatty acids. Retention times of FAME compounds are as follows: 7.74 min for palmitic, 10.590 min for stearic, 10.867 min for oleic, 11.575 min for linoleic and 12.615 min for α-linoleic (linolenic). The retention time for glycerol was 9.02 min, applying the same method. To confirm repeatability, all measurements were performed in triplicate. On 95% confidence interval, the results showed no significant difference.

#### 2.2.7. Macro-Scale Biodiesel Production—Batch Reactor Experiments

Setup for biodiesel production in batch reactors, from edible and waste cooking sunflower oil using Lipolase 100L as catalyst, was previously described by Budžaki et al. [24]. In order to ensure a sufficient amount of methanol in the one-step reaction, the molar ratio of oil to methanol was 1:3.4. All experiments were performed for two days (48 h) with constant stirring (600 rpm). The initial lipase concentration (dissolved in phosphate buffer pH 7.4 (2 experiments) and DES (2 experiments)), was the same in all four experiments (*γ*_Ε,0_ = 0.1 mg/mL). Overview of performed experiments is given in Table 1.

#### 2.2.8. Micro Scale Biodiesel Production—Microreactor Experiments

A glass microreactor with two inlets (length: width: depth = 332 mm: 500 μm: 50 μm with an internal volume of 8.3 μL; Micronit Micro-fluidics B.V., Enschede, Netherlands) was selected for the transesterification process on a micro scale. Total of eight different experiments were performed (Table 1).

In the fifth experimental setup, emulsion which was formed from sunflower oil and enzyme dissolved in buffer (potassium phosphate buffer pH 7.4 (*c* = 0.01 mol/L), in 9:1 ratio), with the addition of emulsifier (SDS), was placed into one syringe, while the second syringe was filled with methanol. In the sixth experiment, sunflower oil was replaced by WCO. In the seventh experiment, enzyme was dissolved in DES (in 1:9 ratio) and mixed with edible sunflower oil and SDS (to form a stabile emulsion). This mixture was placed into one syringe, while the second syringe was filled with methanol. In the eight experiment, experimental conditions were same as in seventh with the exception of edible sunflower oil that was replaced with WCO. Experiments nine (9) to twelve (12) were similar to experiments five (5) to eight (8) but instead of commercial enzyme lipase, lipase produced by solid-state fermentation of by-products from cold-pressing oil and partially purified was used. Syringes were placed on pumps (PHD 4400 Syringe Pump Series, Harvard Apparatus, Holliston, MA, USA) and connected with silica/PTFE tubes to both microreactors. All experiments were performed at 40 °C in order to obtain optimal enzyme activity. A water bath with a heat regulation system (Thermomix 1420, Braun, Germany) was used to secure required temperature.

In order to determine the influence of DES water content on biodiesel yield and volumetric productivity additional set of experiments were performed in a microreactor. As in previous experiments performed with DES, the first experiment in this part of investigation was carried out with anhydrous DES while in other experiments different amounts of water were added to obtain different water content (% *w*/*w*) in the process ranging from 1 to 8%. Those experiments were conducted in a PTFE microreactor (length: width: depth = 1.2 m: 500 μm: 50 μm with an internal volume of 236 μL) for residence time of 30 min.

For all experiments, an oil: methanol: enzyme ratio 10:1.24:1 was kept constant by altering the flow ratios. The influence of total flow rate ranging from *Φ* = 0 µL/min to *Φ* = 200 µL/min) on measurement of fatty acid methyl esters (FAME) formation was monitored. The reaction at the exit of the microreactor was stopped by placing outgoing silicate tubes in an organic solvent (Marmur solution—chloroform: isoamyl alcohol = 24:1, placed in vials and cooled on ice). This combination consequently led to enzyme deactivation [11].

#### 2.2.9. Data Processing

Enzyme operational stability decay rate constant (*k*_d_) was described by first-order kinetics (Equation (1)).

(1) $$\frac{d_{relative\;activity}}{dt}=-k_{d}\cdot relative\;activity$$

The experimental data were the basis for the estimation by nonlinear regression analysis. The least-squares method implemented in the Scientist^®^3.0 software package (Micromath^®^, Saint Louis, MO, USA) was used.

## 3. Results

### 3.1. Transesterification Process in a Batch System

As mentioned earlier, transesterification of oil into biodiesel was conducted in separate batch experiments 1–4 (Table 1). These experiments were conducted because there is no available literature data (with the exception of biotransformation using a commercial enzyme and edible sunflower oil [24]) and in order to ultimately compare the performance of batch and microreactor systems.

A total of four different process conditions were tested. The main goal was to investigate different oil sources (edible and waste sunflower oil) as substrates for biodiesel production, as well as the possibility of using DES as a replacement for an aqueous buffer as the reaction medium [24]. DES was proposed since its composition can provide stability and activity for enzymes such as lipase in deep eutectic mixtures, even under high concentrations of molecules (such as methanol) that can denature the enzyme. In all experiments, for a total time of 48 h, commercial lipase Lipolase 100L was used as a catalyst. In one of our previous experiments [24], this time has been proven as sufficient for biodiesel synthesis by enzymatic transesterification with the high conversion of oil.

The obtained results are presented in Table 2. As it can be observed, the highest biodiesel yield was obtained in experiment 1, where the primary substrate used was edible oil and an aqueous buffer was used as a reaction medium. Somewhat lower yield was noticed in experiment 2, where waste oil was used as a substrate. This can be explained by possible impurities contained in waste oil, which probably had some influence on enzyme activity during the transesterification process.

In the experiments (3 and 4) where DES was used instead of an aqueous buffer, a significantly lower biodiesel yield was noticed. This can be explained by the lack of water in the process (only 0.7% (*w*/*w*) of water was in the reaction medium). According to Merza et al. [25], adding just 1% (*w*/*w*) of water to pure ChCl:Gly DES, thus forming ChCl:Gly:H_2_O DES, higher biodiesel yields can be achieved. Since the commercial enzyme is 73% water, premise was that the water content in Lipolase 100L would be sufficient for enhanced lipase activity. Same researchers report that adding up to 4% (*w*/*w*) of water is preferable in the system to maintain lipase activity.

**Figure 1:**
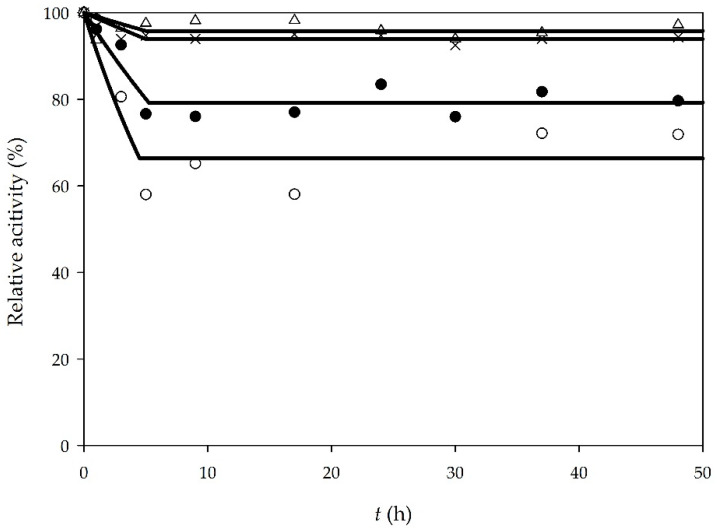
Lipase stability in batch processes (○—edible oil, enzyme dissolved in buffer, ●—waste oil, enzyme dissolved in buffer, ∆—edible oil, enzyme dissolved in deep eutectic solvent (DES), ×—waste oil, enzyme dissolved in DES).

Enzyme operational stability was monitored during all batch processes. In all experiments, samples were taken from the reactor and enzyme activity was measured. Initial enzyme activity was taken as the reference point, and the relative activity for every sample was calculated according to the reference point. The results for enzyme operational stability in all batch experiments are shown in Figure 1. As it can be seen, when an enzyme is dissolved in buffer, there is a decrease in enzyme activity in the first 5 h. There are several reasons why enzyme deactivation occurred, such as mechanical forces, formation of glycerol and water content [23]. Additionally, methanol used in the reaction is one of the representatives of low chain alcohols and it has a significant impact on enzyme activity. According to literature [26], methanol causes the decrease of enzyme activity by stripping bounded water from the enzyme and by penetrating into the enzyme active sight. In our previous papers [11,24], we demonstrated that a higher concentration of methanol (90% (*v*/*v*) solution) completely deactivates the enzyme in 25 h. In this research, the initial concentration of methanol was 30.6 mg/mL, which caused deactivation at the beginning of the process. During the reaction, due to the methanol consumption, the enzyme operational stability did not change significantly, and it was almost constant after the first 5 h.

In order to determine the enzyme operational stability decay rate, Equation (1) was used. For the first 5 h of experiments, if sunflower oil was used as a substrate, the enzyme operational stability decay rate was *k_d_*_sunflower oil/buffer_ = 0.091 ± 0.015 1/h and when WCO was used, the enzyme operational stability decay rate was *k_d_*_WCO/buffer_ = 0.044 ± 0.007 1/h.

Unlike a buffer, DES provided greater stability to lipase (Figure 1). After 48 h of the experiment, lipase activity remained almost the same. The deactivation constants were determined to be *k_d_*_sunflower oil/DES_ = 0.009 ± 0.006 1/h for sunflower oil and *k_d_*_WCO/DES_ = 0.013 ± 0.003 1/h for WCO, respectively. According to literature [27], DES probably “protects” the enzyme by forming hydrogen bonds with methanol. This limits the penetration of the methanol in the enzyme active sight. In addition, choline and chloride DES ions can form hydrogen bonds with the surface residues of the enzyme, leading to better enzyme stability.

### 3.2. Biodiesel Production in A Microreactor

Prior to the microreactor experiment, enzyme operational stability was determined. While the batch processes were carried out in the period of 48 h, continuous processes in microreactors were conducted for a longer period of time. Based on the setup up of the different overall flow rate, 4 days are needed for one experiment to be conducted in a microreactor.

According to literature [11], an enzyme should be stable in buffer for more than five days. For the first four days it remained above 80%, after which the activity dropped significantly, with the measured activity of 30% of the initial activity in the sixth day. The reason for this is probably also methanol which penetrated into the syringe during the microreactor experiments due to back flow. This usually occurs when pumps are stopped, and high pressure is still present in the microchannel.

As mentioned above, in order to find the “ideal” system for biodiesel production by enzymatic transesterification, several microreactor experiments have been set up. The main goal was the intensification of the transesterification process in terms of obtaining similar biodiesel yields for shorter (residence) time in comparison to the batch system. This can be achieved due to the small microchannel size which assures a high surface to volume ratio, which leads to enhanced heat transfer and thus reduced energy demands, faster diffusion as a dominant transport, good process control and high throughput [28].

Based on the chronological order of performed experiments, different comparisons were made, according to the oil source, the reaction medium and enzyme origin. All of the experiments (5–12) were made for same range of residence times (*τ* = 0–30.62 min), so the comparison could be made easier. As main process parameters, biodiesel yield and volumetric productivity were compared for different residence times.

#### 3.2.1. Influence of Oil Origin on Biodiesel Yield and Volumetric Productivity

**Figure 2:**
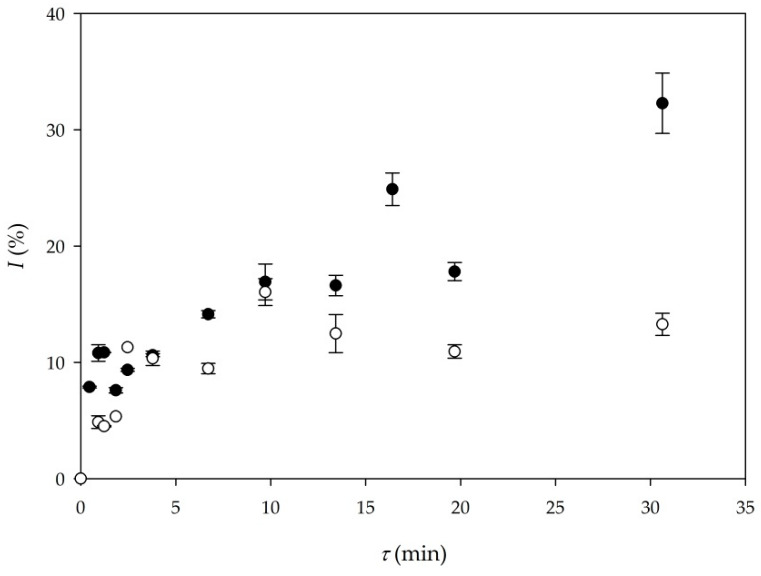
Comparison of biodiesel yield depending on oil origin (○—edible oil (Experiment 5), ●—waste cooking oil (WCO) (Experiment 6)).

First two experiments that were performed in a microreactor were Experiments 5 and 6 (Table 1). Those experiments were performed under the same conditions as batch experiments 1 and 2. In Experiment 5, sunflower oil was used, and in Experiment 6, WCO were used as substrates. The comparison of biodiesel yield for these two experiments is presented in Figure 2. As it can be seen, biodiesel produced from WCO results in higher yield for the same residence time, when compared to biodiesel produced form edible oil. The highest yield of 33% was obtained in the experiment performed with WCO for the longest residence time of 30 min. When it comes to productivity, the comparison between Experiment 5 and Experiment 6 shows that the setup of Experiment 6 (*Q_p_*_6_ = 20.88 kg/ (L∙d)) results in double the volumetric productivity than Experiment 5 (*Q_p_*_5_ = 9.87 kg/ (L∙d)), respectively.

One possible explanation for the highest yield in the experiment with WCO is the cracking of long chains in oil during frying and the resulting shorter chain fatty acids present in the reaction medium during transesterification. The WCO used in this research was analyzed and its composition was compared with fresh sunflower oil. Analysis revealed that WCO had a 4% higher concentration of palmitic acid and a 6% lower concentration of linoleic acid in comparison to fresh sunflower oil. Lipases can then process them faster as a consequence, resulting in higher production yields of biodiesel in the experiment with WCO.

#### 3.2.2. Influence of the Reaction Medium on Biodiesel Yield and Volumetric Productivity

Although the results obtained from batch experiments (Experiments 3 and 4) indicated that when DES is used as a medium for the transesterification process, additional water should be added to the systems to obtain a higher yield reaction. Nevertheless, transesterification in a microreactor with DES as a reaction medium was performed under the same conditions as in batch experiments (Experiments 3 and 4). The first reason was to gain more information about reaction performance in a microreactor and the second one was to test the possibility of simultaneous glycerol removal by using the DES which was present in the system. This possibility of process duality could prove to be a valuable process optimization procedure for biodiesel production in microreactors. In one of our previous papers [29], ChCl:Gly DES was efficiently used for glycerol removal from biodiesel. By using ChCl:Gly (in ratio 1:3.0) based DES, almost all free glycerol was removed from biodiesel. Based on these results, a DES with the same composition was used for biodiesel production. In order to check glycerol removal efficiency in these experiments, the concentration of glycerol was calculated based on the stoichiometry of the reaction (calculated from the FAME concentration for the longest residence time *τ* = 30 min) for the value of 12.58 mg/mL. Analyzing the real sample, the concentration was determined to be 10.68 mg/mL, meaning that 1.9 mg/mL (15.11%) glycerol was removed from the process. The reason for such low extraction efficiency could be the amount of DES in the process. While the amount of DES in the previously performed experiments was 50% (v/v), in this experiment, only 10% (*v*/*v*) of DES was used. Although the obtained efficiency was not enough to satisfy the norms for biodiesel purity, it clearly indicated that with future optimization, an integrated system for biodiesel production and simultaneous glycerol removal on a single microchip could be a solution for further process intensification.

**Figure 3:**
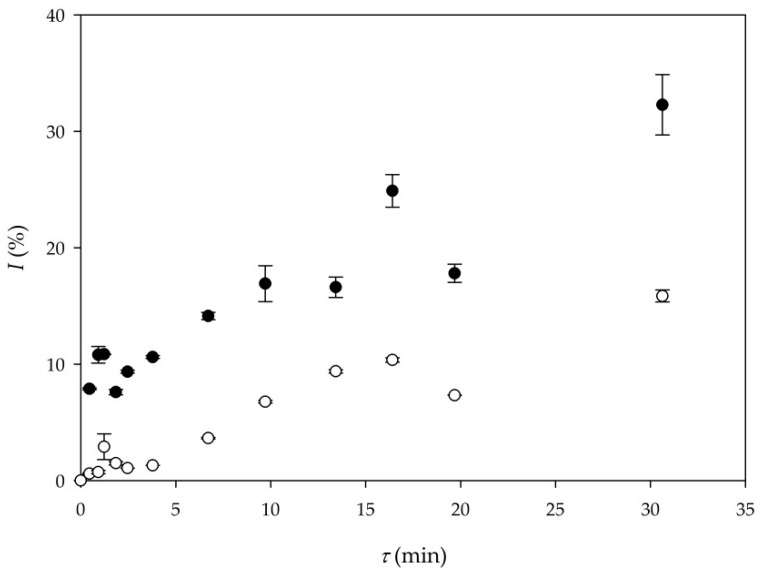
Comparison of biodiesel yield in different reaction mediums (●—buffer as a reaction medium (Experiment 6), ○—DES as a reaction medium (Experiment 8)).

Figure 3 shows the comparison between yields obtained in Experiment 6 (buffer) and Experiment 8 (DES).

As it can be seen in Figure 3, higher biodiesel yield was again obtained in the experiment where a buffer was the reaction medium (Experiment 6). In the experiment performed with a DES as the reaction medium, approximately 15% of the biodiesel yield was obtained for the residence time of 30 min. The obtained volumetric productivity in Experiment 6 (*Q_p_*_6_ = 20.88 kg/ (L∙d)) was almost three times the volumetric productivity of Experiment 8 (*Q_p_*_8_ = 7.35 kg/ (L∙d)), respectively.

Although the experiment performed with DES resulted in approximately 15% biodiesel yield (*τ* = 30 min), this is significantly higher in comparison with biodiesel yield obtained in batch experiments, where biodiesel yields were only 6% (Experiment 3) and 5% (Experiment 4), respectively. In addition, batch experiments were performed for 48 h, which also favors microreactor experiments. This indicates that using anhydrous DES in microreactor systems significantly increases productivity, which is visible in Table 3 if comparing Experiments 3 and 4 to Experiments 7 and 8. Furthermore, adding water content up to 4% (*w*/*w*) [26], could provide additional improvement in biodiesel yield for the same residence time.

#### 3.2.3. Influence of Enzyme Origin on Biodiesel Yield and Volumetric Productivity

**Figure 4:**
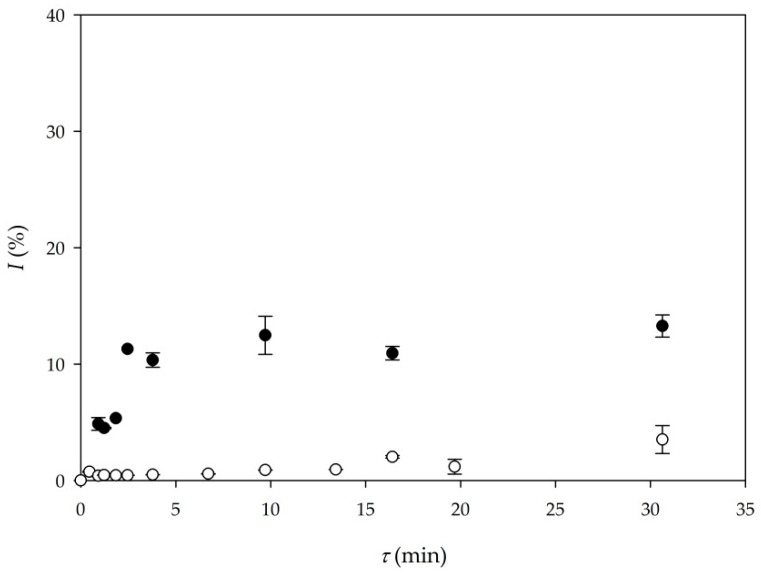
Comparison of biodiesel yield according to lipase origin (●—commercial lipase Lipolase 100L (Experiment 5), ○—purified lipase produced by solid-state fermentation (Experiment 9)).

In order to make the overall process more economical, the application of lipase, produced by solid-state fermentation of *Thermomyces lanuginosus*, on by-products from cold-pressing oil production and partially purified, was used for biodiesel production. Results were compared with the results obtained for biodiesel production by transesterification catalyzed by commercial lipase. As it can be seen form Figure 4, there is a significant difference in biodiesel yield when commercial lipase and produced and purified lipase [22] were used. The main reason behind this is the much larger initial activity of the commercial enzyme (around 200 times higher) in comparison to the purified enzyme. At this point, without additional optimization of purification steps, purified enzyme is not suitable for biodiesel production. A new approach in making the process more economical would be the immobilization or the recirculation of commercial lipase. Furthermore, a productivity-based comparison between Experiment 5 and Experiment 9 shows that the setup of Experiment 5 (*Q_p_*_5_ = 9.87 kg/ (L∙d)) provides much higher volumetric productivity than Experiment 9 (*Q_p9_* = 1.63 kg/ (L∙d)), respectively, mostly due to the much higher initial lipase activity.

### 3.3. Influence of DES Water Content on Biodiesel Yield and Volumetric Productivity

Additional experiments were performed to explore the possibility of using DES as adequate replacement for a buffer in terms of higher biodiesel yields. Experiments were performed with different water content ranging from 1 to 8% (*w*/*w*). Since all the previously conducted experiments have shown the highest biodiesel yield for residence time of 30 min, all experiments with different DES water content were performed for that residence time. To confirm the repeatability of experiments, the experiment with 0% of water (*w*/*w*) was compared to Experiment 7, performed at same initial conditions and the results have shown strong agreement. If water was added in the reaction medium biodiesel yield and consequently volumetric productivity, rises all the way up to 4% (*w*/*w*) of added water (Table 3). This agrees with the study conducted by Merza et al. [25], since the water content of 4% (*w*/*w*) was also optimal for enzymatic production of biodiesel by transesterification. If the water content was above 4% (*w*/*w*), biodiesel yield and volumetric productivity start to decrease and for 8% (*w*/*w*) of water, both biodiesel yield and volumetric productivity where lower than in the experiment without the addition of water. This is probably due to hydrolysis being more pronounced in the reaction system with higher water content.

### 3.4. Comparison of Different Systems for Biodiesel Production

The comparison of biodiesel production using different systems has clearly shown the guidelines for the forthcoming research. In Table 4 biodiesel yield and volumetric productivity obtained in a batch and in a continuous microreactor have been shown. The highest volumetric productivity achieved in a batch process (Experiment 1) has significantly lower productivity in comparison with the highest volumetric productivity obtained in a microreactor experiment (Experiment 6). These results show that enzymatic transesterification in microreactors is a viable option, even WCO was used as a substrate. Unfortunately, biodiesel yield and productivity obtained in this research are significantly lower if compared with earlier research of Šalić et al. [11] (Experiment 13, Table 4). The main reason for it is the high methanol excess (28-fold higher than stoichiometric one) used in Experiment 13. Namely, the methanol concentration in Experiment 6 was 30.60 mg/mL, while in the Experiment 13, methanol concentration was 836.97 mg/mL. Microchannel width in Experiment 13 was 1 mm, which is unfavorable in comparison with the microchannel width in Experiment 6 in terms of mass transfer. Still, the high methanol excess was the dominant reason for better results in a bigger microchannel. If the results of this research were compared with experiments performed by Gojun et al. [30] (Experiment 14), the results go in the favor of the smaller microchannel width. Namely, in Experiment 14, the microchannel width was 250 μm, which leads to four-fold faster diffusion in this microreactor system. In addition, in Experiment 14, the microreactor was fed with three inlets. Oil, enzyme suspended in buffer and methanol were fed separately. In Experiments 5–12, oil and enzyme with the addition of emulsifier were fed as one process stream and methanol was the second inlet stream. Faster diffusion and absence of emulsifier could be a reason for better results obtained in Experiment 14.

Lastly, the comparison of the experiments involving a DES as the reaction medium shows some interesting insights on whether these systems have good future applications. Experiments 7 and 8 were conducted with the commercial enzyme in a DES as the reaction medium. Those experiments provided biodiesel yield of 12–15% (Table 4), which is less when compared to Experiment 15, performed by Merza et al. [25], where biodiesel yield was up to 40%. However, with calculated productivity of these systems (shown in Table 4), Experiments 7 and 8 conducted in microreactor systems provide 3–4 times higher productivity than Experiment 15. These results justify the usage of a DES as the reaction medium, with increasing the water as a possible enhancement.

All in all, the direction for future experiments should definitely be the usage of waste oil as substrate, methanol in higher surplus than conducted in this research and smaller channel width. All the data shown indicates that channel width smaller than 1 mm is suitable for process intensification.

## 4. Conclusions

Biodiesel production by enzymatic transesterification has been conducted successfully. This study represented a total of twelve different experiments, eight of which were conducted in microreactors. The importance has been put on developing the “ideal” microsystem, the one which will provide the highest biodiesel yield for the shortest residence time. Results show that enzymatic transesterification in microreactors is a viable option for using waste oil as a primary substrate. The highest yield of 32% for residence time *τ* = 30 min was obtained in the microreactor system with an emulsion of waste oil and commercial enzyme suspended in a water buffer. This knowledge may prove rather important in future experiments and in process optimization, where the next steps are kinetic studies and mathematical modeling. Furthermore, even though results obtained with DES were not as good as those obtained in a buffer medium, this opened up another research area. While DESs have already been confirmed as great extraction mediums, their role in synthesis still needs further investigation. Future experiments will provide momentum for developing the multi-purposed significance of DES.

## Figures and Tables

**Table 1:** Experimental setup and different reaction conditions during oil transesterification catalyzed by lipase performed in a batch reactor and in a microreactor system.

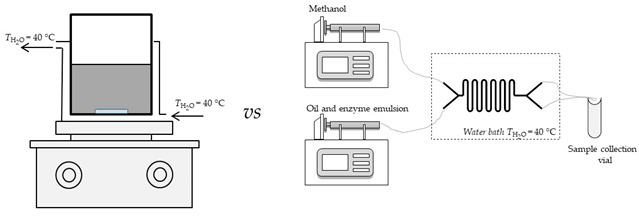						
	Oil Type		Enzyme		Solution	
	Sunflower Oil	WCO	Commercial	Partially Purified	Buffer	DES
**Batch Reactor**						
Experiment 1	+		+		+	
Experiment 2		+	+		+	
Experiment 3	+		+			+
Experiment 4		+	+			+
**Microreactor**						
Experiment 5	+		+		+	
Experiment 6		+	+		+	
Experiment 7	+		+			+
Experiment 8		+	+			+
Experiment 9	+			+	+	
Experiment 10		+		+	+	
Experiment 11	+			+		+
Experiment 12		+		+		+

**Table 2:** Comparison of biodiesel yield obtained in batch experiments.

	Time, h	Biodiesel Yield, %
Experiment 1	**48**	**91**
Experiment 2	**48**	**70**
Experiment 3	**48**	**6**
Experiment 4	**48**	**5**

**Table 3:** Comparison of biodiesel yield and volumetric productivity for different DES water content in the transesterification reaction catalyzed by lipase.

Water Content (% *w*/*w*)	*τ* (min)	*I* (%)	*Q_p_* (kg/ (L∙d)
0	30.62	12.38	5.74
1	30.62	13.31	6.17
2	30.62	16.81	7.79
4	30.62	31.22	14.47
6	30.62	23.03	10.68
8	30.62	10.62	4.92

**Table 4:** Comparison of the oil transesterification process performed in a batch and in different types of microreactors.

Experiment	*t* (h)	*I* (%)	*Q_p_* (kg/ (L∙d)	Reference
1	48	91.34	0.45	This research
2	48	70.22	0.35	
3	48	6.44	0.03	
4	48	5.23	0.02	
	***τ* (min)**			
5	30.62	13.26	9.87	
6	30.62	32.28	20.88	
7	30.62	12.42	5.76	
8	30.62	15.85	7.35	
9	30.62	3.51	1.63	
10	30.62	20.08	9.31	
11	30.62	1.76	0.81	
12	30.62	5.45	2.53	
13	30	97.81	69.88	[11]
14	19.8	32.72	35.42	[30]
	***t*** **(h)**			
15	8	40	1.78	[26]
